# Editorial: Predictive Mechanisms of the Cerebello-Cerebral Networks

**DOI:** 10.3389/fncel.2019.00549

**Published:** 2019-12-10

**Authors:** Mario U. Manto, Aasef G. Shaikh

**Affiliations:** ^1^Department of Neurology, CHU-Charleroi, University of Mons, Mons, Belgium; ^2^Movement Disorders Division, Neurological Institute, University Hospitals Cleveland, Cleveland, OH, United States; ^3^Departments of Neurology and Biomedical Engineering, Case Western Reserve University, Cleveland, OH, United States; ^4^Neurology Service and Daroff-Dell'Osso Ocular Motility Laboratory, Louis Stokes Cleveland Medical Center, Cleveland, OH, United States

**Keywords:** cerebellum, behavior, model, cognition, memory

Two apparently distinct tasks, action and perception, are closely interlinked from the physiological and philosophical standpoints. In general terms, the action is comprised of interpreting, in motor context, what had been perceived, modulating the future motor commands to make the consequence (i.e., perception) favorable, and program the movement for seamless performance. Given their interdependence, nature has provisioned many strategies to facilitate flawless interaction between the action and perception.

One of the fundamental needs for the smooth action is precise movement and its online correction. The crystalline architecture of the cerebellum, its huge number of neurons and its multimodal connectivity had historically fascinated the neuroscience community by its highly responsive ability to adapt movements and correct errors. The cerebellum is famously known as the brain's learning machine. The advent of the concept of internal model in the 1970s by Franscis and Wonham promoted the idea that the cerebellum is not only the learning machine, but it also excels in machine learning (Francis and Wonham, 1976). The principle of internal model emphasizes that the motor system is controlled by the constant communication between the conveyer of action (i.e., the motor plant—the muscles and joints) and the executor of action (i.e., the controller—the cerebral cortex). The interaction between the conveyer and executer through multiple cerebello-cerebral running in parallel is facilitated and modulated by the intervening process, the internal models, that rely on the internal and external information. The motor command from the executor, when it reaches the conveyer, also provides a “carbon copy” to cerebellar cortex and nuclei, also known as the efference copy. Latter is utilized by the conceptual mechanism called the forward model that subsequently predicts the consequence (i.e., the predicted outcome). The predicted consequence is then utilized by the brain to refine the future commands. The fundamental question that remains is—how does the brain predict the future? Contemporary literature has suggested the fundamental role of the cerebellum in prediction and facilitating the forward model. In this facilitation the cerebellum utilizes the prior experiences and runs inbuilt algorithms, just like machine learning, to predict consequences that are then linked to the action. After three centuries of experimental and clinical experiments, cerebellar research has jumped from motor control to cognition, behavior, and evolution of homo sapiens. The frontiers topic “Predictive Mechanisms of the Cerebello-Cerebral Networks” highlights a unique collection of papers offering a succinct understanding of cerebellar predictive mechanisms of forward model utilizing various contexts and neurological systems.

In a comprehensive review Molinari and Masciullo summarizes how predictions utilizing the forward model are implemented in an important cerebellar function of determining right sequence of desired task. Complementing review by Popa and Ebner further emphasizes the mutually beneficial dependence of cognitive and motor tasks that are necessary for effective prediction.

Four original research papers describe the concept of forward model in mutually exclusive experiment models and neurological systems. In non-human primate ocular motor system Kim et al. discovered the neural correlate of the forward model vs. those of the motor commands by examining the neural responses during smooth pursuit eye movements. The authors conclude that predictive information is constructed from the motor command information and the construction of such predictive signal happens at the level of cerebellar cortex. The studies in ocular motor system in non-human primate was accompanied by the paper by Kakei et al. where physiological analysis of limb movements was performed in the ataxia victims. By utilizing a non-invasive computational method of analyzing the movement kinematics the authors identified two components of the arm movements—one encoding the velocity and position of the moving object while another the position control. The cerebellar patients had abnormal measure of the first component suggesting impaired accuracy of the predictive control.

The subsequent study by Sheu et al. switched gears, examining the role of cerebellar prediction and forward model in verbal working memory. The authors demonstrated a predictive process that provides the supervision for the cerebellar non-motor function, particularly predictive role in phonological loop in verbal working memory.

Clausi et al. focused on the role of cerebellum in the social cognition, on the “theory of mind”—the process involving the emotions, intentions, and beliefs. The authors found that the cerebellum may implicitly match the external information with the internal presentation, for example linking the facial expression and corresponding mental state. It was suggested that cerebellum constructs the internal models of the mental processes during social interaction where the prediction of sequential events helps anticipate the other person's behavior.

Finally, in a hypothesis paper, Vandervert extended the cerebellar role of prediction-based sequence detection on human evolution and relentless advancement of the culture. This is a particularly challenging concept with social and cultural implications for humans. Cerebellum appears as a highly modifiable hard disk refining behavior (motor and social) to adapt to a constantly changing world. Physiology and philosophy are intimately linked again. Cerebellar sequencing detection participates in the evolution of culture, language and stone-tool technology, landmarks of Homo sapiens.

Ultimately this Research Topic heightened our understanding of the well-rounded application of the cerebro-cerebellar predictive mechanism featuring the forward model. The consequences of malfunctioning forward model can be grave, and they can affect a wide range of systems from motor functioning to perception, cognition, and behavior. Specific targeted approaches to rehabilitate the predictive mechanisms in the diseased states, either by means of drugs, non-invasive neuromodulation or combinations are needed. The contribution of the cerebellum in numerous brain functions is growingly recognized, with implications in numerous prevalent neurological and neuropsychiatric conditions including essential tremor, Parkinson's disease, multiple sclerosis, autism spectrum disorders and schizophrenia (Miterko et al., 2019). We predict that the future of cerebellar research will just keep spreading.

## Author Contributions

MM and AS interacted and validated the final version of the manuscript.

### Conflict of Interest

The authors declare that the research was conducted in the absence of any commercial or financial relationships that could be construed as a potential conflict of interest.
